# Editorial: Bridging the gap between integrative neuroscience and translational neuroscience

**DOI:** 10.3389/fnint.2023.1296701

**Published:** 2023-10-05

**Authors:** Elias Manjarrez, Giulia Curia, Katinka Stecina, Alejandro Lopez Valdes

**Affiliations:** ^1^Instituto de Fisiología, Benemérita Universidad Autónoma de Puebla, Puebla, México; ^2^Department of Biomedical, Metabolic and Neural Sciences, University of Modena and Reggio Emilia, Modena, Italy; ^3^Department of Physiology and Pathophysiology, Rady Faculty of Health Sciences, University of Manitoba, Winnipeg, MB, Canada; ^4^Department of Electronic and Electrical Engineering, Trinity College Dublin, Dublin, Ireland; ^5^Global Brain Health Institute, Trinity College Dublin, Dublin, Ireland; ^6^Trinity College Institute of Neuroscience, Trinity College Dublin, Dublin, Ireland; ^7^Trinity Centre for Biomedical Engineering, Trinity College Dublin, Dublin, Ireland

**Keywords:** neurostimulation devices, transcranial magnet stimulation, electrical stimulation, neuroprostheses, visual imaginary, Allen Institute for Brain Science

Integrative neuroscience involves investigations into the sensory, motor, and cognitive systems. In contrast, translational neuroscience moves fundamental knowledge and interdisciplinary discoveries into clinical practice or applications to benefit human health. Bridging the gap between both fields could profoundly impact our comprehension of brain function and clinical applications (Figure 1). Specifically, the five articles presented here address some of the current interests and gaps in knowledge. This Research Topic covers three papers about tactile, vestibular, and motor systems in the context of electrical or magnetic stimulation. Moreover, the topic covers two other articles on the cognitive processes of people with blindness and neuronal informatics to understand the neurological impact of COVID-19.

**Figure 1:**
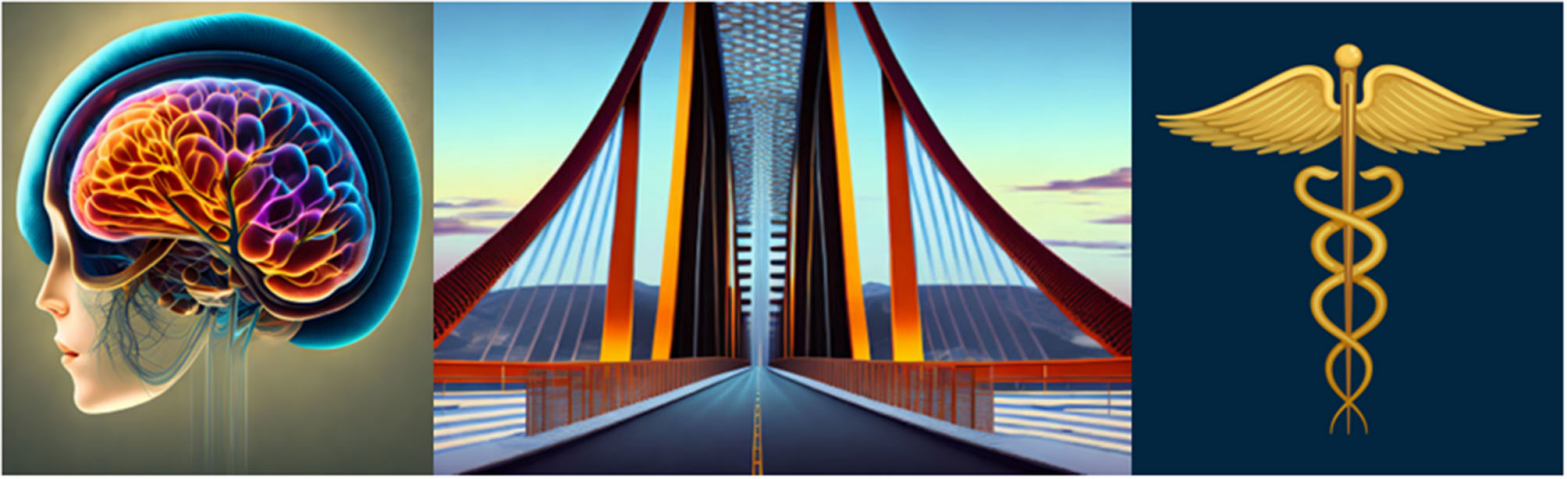
The images highlight the importance of connecting integrative and translational neuroscience to enhance our understanding of brain function and its clinical applications. This Research Topic of five articles focuses on bridging the gap between these two areas of study. The first two artistic representations were produced with consent from www.deepdreamgenerator.com using a paid “energy pack” for image creation. The medical symbol image was obtained from https://www.freepik.com/free-vector/flat-medical-symbol_28903697.htm#query=medical%20.

The first two papers in this Research Topic have reported new methods to push the limits of precision in brain and spinal cord neurostimulation. They are relevant because non-invasive neurostimulation of the brain, peripheral nerves, and the spinal cord has unprecedented clinical interest. In this context, Fujiki et al., introduced the “quadripulse theta burst transcranial magnetic stimulation” (QTS), a new way to overcome failure rates in producing motor evoked potentials by transcranial magnetic stimulation. These authors employed a similar stimulation pattern as in previous studies (Szelényi et al., 2007; Deletis and Sala, 2008; Hamada et al., 2013; Tsutsui et al., 2015; Deletis and Fernández-Conejero, 2016; Jung et al., 2016). This QTS method amplified motor evoked potentials (MEPs) a thousand-fold, resulting in four independent MEPs 20 ms after each burst onset. The significance of this study is the potential use of QTS for motor palsy functional evaluation after intracerebral hemorrhage. In the same context of new neurotechnology developments, targeting the desired cluster of neurons by electrical stimulation is also challenging in the spinal cord when using non-invasive electrical stimulation. Here, Chandrasekaran et al. introduced a novel, highly flexible spinal electrode array for cervical dorsal root stimulation employed in people with motor complete spinal cord injury. This targeted stimulation increased volitionally generated force and tactile sensations within a 6–8-week period, but only in particular muscles showing discernable force production during the pre-intervention assessment. These results are comparable to those found by other authors (Freyvert et al., 2018; Inanici et al., 2021; Huang et al., 2022).

The third paper by Soto et al. is a review covering the development of new active vestibular implantable devices to regain or modulate specific systemic functions. Like cochlear implants, pacemakers, or deep brain stimulators, the vestibular neuroprostheses are a strong example of how bridges between integrative and translational neuroscience are needed to achieve beneficial breakthroughs that will impact the lives of individuals. Vestibular dysfunction affects over 1.8 million people worldwide (Chow et al., 2021), influencing more than posture and balance. For instance, vestibular information propagates to various neural systems via the reticular formation, regulating alertness and autonomic function (Lane et al., 2019). In this Research Topic, the authors present a comprehensive review of the state-of-the-art in the development and testing of various prototypes of vestibular implants. Their review covers the 30-year journey from the first studies on external vestibular system stimulation to the recent successful human implantation studies. This review paper also highlights the challenges of producing vestibular responses, considering the complex interaction with hearing and the otolithic organs (Ramos de Miguel et al., 2020).

The fourth article by Ilic et al. deals with visual imagery in dreams of congenitally blind people. This theme is controversial (Andrade, 2021). It has been shown that the lack of sensory stimulus in one system could be rescued by increased sensory-motor stimulation of other sensory systems. In addition, stimulation of other sensory modalities accelerates the development of the visual cortex, even without visual deprivation. This evidence suggests that the visual system may contribute to oneiric visual imagery-like perceptions, even in blind subjects. The review by Ilic et al. in this Research Topic analyses studies on the presence and nature of visuospatial imagery in dreams of blind people to elucidate how blind people “see”, whether they may recreate visuospatial imagery via sensory substitution, and whether they can dream in images. At the neurophysiological level, neuroimaging and sensory substitution studies suggest that the “blind” occipital cortex may be able to integrate non-visual sensory inputs, generating visuospatial impressions and enabling the development of a typical spatiotemporal organization of early visual areas even in the life-long absence of vision. This could explain the ability of some congenitally blind individuals to draw symbolic representations of various visual images in striking likeness to those drawn by normally sighted. Therefore, elucidating the mechanistic nature of visual impressions could open new translational possibilities for treating these neuro-disabilities.

Finally, Mesmoudi et al. presented another interesting research covering the gap between integrative neuroscience and translational neuroscience in the context of the recent global health crisis of COVID-19, in which the discovery of ACE2 receptor mechanisms played a fundamental role in understanding this sickness. They employed data on mRNA expression levels of genes provided by the Allen Institute for Brain Science. Moreover, the localization of brain functions was provided by the LinkRbrain platform. These authors investigated which cognitive and sensorimotor functions are associated with the brain regions where ACE2/TMPRSS2 is overexpressed, hypothesizing that the infection might particularly affect them. The results show that central regions specific to ACE2 and MPRSS2 were localized in the brain stem, the subcortical, the orbitofrontal, and some occipital areas (see also Chen et al., 2021).

In conclusion, these five papers, taken together, emphasize that we must bridge the gap between knowledge and practice, between theory and therapy. Thus, the synergy between integrative and translational neuroscience involving new neurotechnological developments could serve as the bridge that will lead us to a future where neurological conditions are better comprehended and more effectively treated. As we persist in exploring the frontiers of the brain, let us bear in mind that we could discover the key to unlocking the full potential of neuroscience for the benefit of humanity through the fusion of these two established disciplines.

## Author contributions

EM: Writing—review and editing. GC: Writing—review and editing. KS: Writing—review and editing. AL: Writing—review and editing.
